# Adult attachment and trait anxiety among Chinese college students: A multiple mediation model

**DOI:** 10.3389/fpubh.2022.912128

**Published:** 2022-08-15

**Authors:** Xu Li, Peizhen Sun, Li Li

**Affiliations:** ^1^Center for Counseling and Psychological Services, Guangdong University of Petrochemical Technology, Maoming, China; ^2^Department of Psychology, School of Education Science, Jiangsu Normal University, Xuzhou, China

**Keywords:** adult attachment, self-esteem, perceived social support, security, trait anxiety

## Abstract

The present study discusses the relationship between adult attachment and trait anxiety of Chinese college students based on the “internal working models” theory. Six hundred and seventy-two valid data were collected using a self-reported questionnaire. The mediating roles of self-esteem, perceived social support, and security in the relationship between adult attachment orientations (anxiety and avoidance) and trait anxiety were investigated using the structural equation model and bootstrap methods. The results showed that: self-esteem and security play mediating roles in the relationship between attachment anxiety and trait anxiety. self-esteem, perceived social support, and security play mediating roles in the relationship between attachment avoidance and trait anxiety. In addition, there were different mediation mechanisms in the correlation between different attachment dimensions and trait anxiety. This study provided empirical data for exploring the formation and maintenance of trait anxiety in college students and had a specific significance for the early prevention and clinical intervention of anxiety-related disorders.

## Introduction

The further development of COVID-19 has hurt people's mental health, leading to anxiety and depression among some college students (1). Trait anxiety reflects the relatively persistent and stable differences in individual anxiety tendencies and is a critical predisposition factor for anxiety-related disorders (2). College students leave their families of origin, come to the university campus, and establish an attachment mode dominated by adult attachment. Therefore, exploring the relationship and mediating mechanism between adult attachment and trait anxiety in the COVID-19 pandemic is essential. The results of this study had implications for the prevention and intervention of anxiety-related disorders among college students.

Attachment, an intimate relationship between individuals and caregivers formed in the early years, is significant to individual psychological development and emotional regulation. The internal work model (IWMs) is an individual's internal mental representation and cognitive schema about self and others formed in early parent-child interaction (3). This cognitive schema works on an individual's early attachment objects and can extend to new interpersonal relationships with age growth (4). With age, the individual's different cognitive schemata about self and others develop into different adult attachment orientations and become an essential basis of individual psychological security (5). Brennan et al. (6) divided adult attachment orientations into two dimensions of “attachment anxiety” and “attachment avoidance” based on the differences between self-representation and other-representation in IWMs. Individuals with higher levels of attachment anxiety adopt “hyperactivating strategy,” while individuals with higher levels of attachment avoidance adopt “deactivating strategy” (7). “Hyperactivating strategy” is a kind of emotion regulation strategy to obtain security by constantly trying to establish relationships with others. “Deactivating strategy” is a kind of emotion regulation strategy to avoid emotional investment by suppressing the need for attachment through self-protection.

Emotional dysregulation is an important factor affecting individuals' trait anxiety (8). A few studies on the relationship between adult attachment and anxiety levels have also proved that emotional dysregulation was a key mechanism linking attachment and state anxiety [e.g., (9)]. However, there are few studies on the effect of adult attachment on individual trait anxiety. Therefore, this study intends to establish the following hypotheses based on IWMs and adult attachment emotion regulation strateies: attachment anxiety and avoidance correlated with trait anxiety (H1a); Attachment anxiety and attachment avoidance have different mechnisems in predicting trait anxiety (H1b).

Self-esteem is the core self-evaluation and emotional experience of individuals. Greenberg et al. (10) terror Management theory regards self-esteem as a universal buffer for dealing with stress and fear in human evolution. The self-representation formed by individuals in interacting with the attachment object is the basis for developing individual self-esteem (11). Individuals with secure attachment have the highest level of self-esteem compared to insecure attachment (12), but research on the relationship between insecure attachment and self-esteem has been inconsistent. A study based on a sample of American college students found that attachment anxiety was negatively correlated with self-esteem after controlling for relevant variables. Still, attachment avoidance was not significantly correlated with self-esteem (13). This result is inconsistent with the study of Li and Zheng (14), who found that attachment anxiety and avoidance are negatively correlated with self-esteem in Chinese culture. In addition, it has been found that self-esteem is an essential factor affecting trait anxiety, and childhood parental bondings can affect trait anxiety in adulthood through individual self-esteem (15). Therefore, this study proposed the hypothesis: self-esteem mediates the relationship between attachment anxiety and trait anxiety (H2a). In addition, considering that this study was conducted in Chinese culture, we also hypothesized that self-esteem does not mediate the relationship between attachment avoidance and trait anxiety (H2b).

As an essential psychological resource possessed by individuals, social support plays a buffering effect between external pressure and individual mental health (16). Perceived social support refers to an individual's expectation and evaluation of social support or belief that it is possible to obtain it (17). Previous studies have shown that perceived social support, as a stable feature with individual differences, is closely related to personal subjective feelings and emotional satisfaction and affects the mental health level of college students (18). Furthermore, Calvo et al. (19) found that attachment anxiety and avoidance may negatively influence perceived social support. Therefore, this study proposed that perceived social support mediates the relationship between attachment orientations (anxiety and avoidance) and trait anxiety (H3a and H3b).

According to attachment theory, the acquisition and satisfaction of security is a critical threshold to trigger individual emotion regulation behavior (5). Security is an individual's strong sense of coping with all kinds of possible dangers, expressed as the sense of prediction, determination, and control of life (20). Insecurity is the typical personality basis of various neuroses, and many neurotic behaviors of human beings are caused by the excessive compensatory pursuit of security (21). When the level of security is reduced, individuals feel that they are facing more significant risks and may be unable to cope with them, resulting in worry and nervousness. Attachment is the basis for individual security formation and change (22). Meanwhile, existing studies have shown that self-identification (22) and perception of external support (23) are important sources of individual security. Therefore, we propose the following hypotheses: security mediates the relationship between attachment orientations (anxiety and avoidance) and trait anxiety (H4a and H4b); self-esteem and security play serial roles in the relationship between attachment orientations (anxiety and avoidance) and trait anxiety (H4c and H4d); perceived social support and security play serial roles in the relationship between attachment orientations (anxiety and avoidance) and trait anxiety (H4e and H4f).

**Figure 1:**
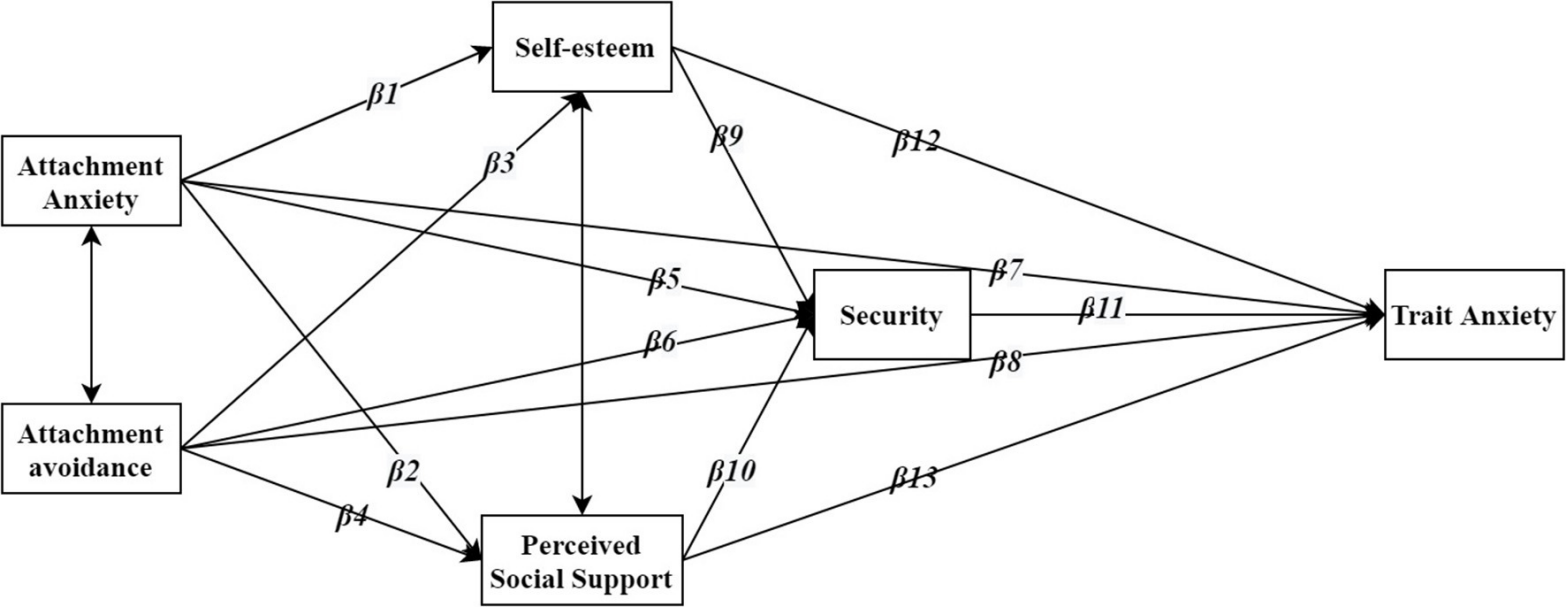
Research hypotheses framework.

Based on the review of existing research and IWMs theory, this study established a multi-mediating model based on these assumptions to investigate the relationship between adult attachment orientations (anxiety or avoidance) and trait anxiety. The hypothesis model is as follows (see Figure 1).

## Materials and methods

### Participants

During the COVID-19 pandemic, we recruited 693 undergraduates from two universities in Jiangsu and Guangdong province using a cluster random sampling method. Participants voluntarily completed the paper-and-pencil version during the class break. This study was conducted under applicable ethics regulations. Participants signed the informed consent and were given gifts to those who completed the survey. After removing 15 uncompleted and six regular questionnaires, 672 valid questionnaires were collected, with an effective rate of 97%. The distribution of the subjects was as follows: 360 male and 312 female; Age ranged from 17 to 23 years (*M* = 19.39; *SD* = 1.05). Among them, 326 freshmen (48.5%), 231 sophomores (34.40%), and 115 juniors (17.10%).

### Measures

#### Adult attachment

Adult attachment was measured using the revised version of the Experiences in Close Relationships Scale (ECR-R) developed by Fraley et al. (24). The ECR-R has 36 items and uses a 7-point Likert scale (1 = “strongly disagree,” 7 = “strongly agree”) to measure the two dimensions of “attachment anxiety” (e.g., “I worry that I won't measure up to other people.”) and “attachment avoidance” (e.g., “I tell my partner just about everything.”). This scale has been widely adopted and has demonstrated good reliability in Chinese participants (25). In this study, the internal consistency coefficients of the Chinese version were 0.89 (attachment anxiety) and 0.87 (attachment avoidance), respectively.

#### Self-esteem

Self-esteem was measured using the self-esteem Scale (SES) developed by Rosenberg (26). The scale is a single-dimensional self-rating scale, consisting of 10 items (e.g., “I take a positive attitude toward myself.”), using Likert scale 4 (1 = “strongly disagree”, 4 = “strongly agree”). This scale has been widely adopted and has demonstrated good reliability in Chinese participants (27). In this study, the internal consistency coefficient was 0.86.

#### Perceived social support

Perceived social support was measured using the published Perceived Social Support Scale (PSSS) (17). The scale consists of 12 self-rated items on a 7-Likert scale (1 = “strongly disagree,” 7 = “strongly agree”). The Chinese version is divided into three subscales of family support (e.g., “My family really tries to help me.”), friend support (e.g., “My friends really try to help me.”), and other support (e.g., “There is a special person with whom I can share my joys and sorrows.”). The Chinese version demonstrated good reliability in Chinese participants (28). In this study, the α coefficients of “family support,” “friend support,” and “other support” subscales were 0.84, 0.87, and 0.86, respectively.

#### Security

The security questionnaire (SQ) prepared by Cong and An (20) was used to evaluate the safety score of neurotic patients and normal people. The scale has 16 items, using a 5-point Likert scale (1 = “very consistent”, 5 = “very inconsistent”), including two dimensions of “interpersonal security” (eight items; e.g., “I never dare to volunteer my opinion”) and “sense of definite control” (eight items; e.g., “I always worry that something bad will happen”). In this study, the internal consistency coefficients of the “interpersonal security” and “sense of definite control” were 0.77 and 0.78, respectively.

#### Trait anxiety

Trait anxiety was measured using the trait anxiety subscale (TAI) of the State-Trait Anxiety Scale (STAI) developed and revised by Spielberger (29). The TAI subscale includes 11 items of negative emotion and nine items of positive emotion (e.g., “I feel pleasant”), using a 4-point Likert scale (1 = “almost never”, 4 = “almost always”). TAI subscale is mainly used to evaluate stable personality traits such as anxiety and tension. This TAI scale has been widely adopted and has demonstrated good reliability in Chinese participants (30). In this study, the internal consistency coefficient of the revised Chinese version was 0.84.

### Procedures

The data analysis of this study was carried out in two steps. The first step was to conduct a descriptive statistical analysis of variables and test the correlation between variables and gender differences. Second, structural equation modeling was used to examine further the relationship between adult attachment dimensions and trait anxiety and the mediating roles of self-esteem, perceived social support, and security. Maximum likelihood estimation (ML) and bootstrap method with bias correction were used to establish the structural equation model and evaluate the mediating effects. Item parceling method was used to solve the problem of too many observation indicators of individual latent variables. R 4.0 and *Mplus*8.0 were used for all statistical processing in this study.

## Results

### Descriptive statistics

The means, standard deviations, and correlations of all variables are presented in Table 1. The skewness coefficient of the data is between −0.59 and 0.50, and the kurtosis coefficient is between −0.58 and 0.61, indicating that the data meet the normality requirement for structural equation analysis (31). The variance inflation factor (VIF) is between 1.40 and 1.90, indicating no multicollinearity problem in the data. Independent sample *T*-test results showed that there were gender differences in attachment avoidance [*t*_(670)_ = 3.80, *p* < 0.001] and perceived social support [*t*_(670)_ = −3.23, *p* < 0.005]. These differences indicated that women have higher attachment avoidance levels and lower social support than men. Therefore, gender as the control variable is included in the model analysis in the following mediation effect analysis.

**Table 1:** Descriptive statistics and correlations among the variables.

**Variables**	**1**	**2**	**3**	**4**	**5**	**6**
Attachment anxiety						
Attachment avoidance	0.39^***^					
Self-esteem	−0.38^***^	−0.42^***^				
Perceived social support	−0.28^***^	−0.56^***^	0.42^***^			
Security	−0.47^***^	−0.41^***^	0.61^***^	0.43^***^		
Trait anxiety	0.53^***^	0.37^***^	−0.59^***^	−0.39^***^	−0.62^***^	
*M ± SD*	61.32 (18.85)	51.57 (15.80)	30.62 (4.89)	62.50 (13.44)	55.39 (10.42)	42.14 (7.96)

*N = 672*.

*** *p < 0.001*.

### Common method biases test

Exploratory factor analysis without rotation was performed using Harman single factor test on all measurement items. The results showed that there were six factors with characteristic roots >1, among which the first factor accounted for 19.15% of the total variation, far less than the critical value of 40%, indicating that there was no serious common method bias in this study (32).

### Mediation analyses

**Figure 2:**
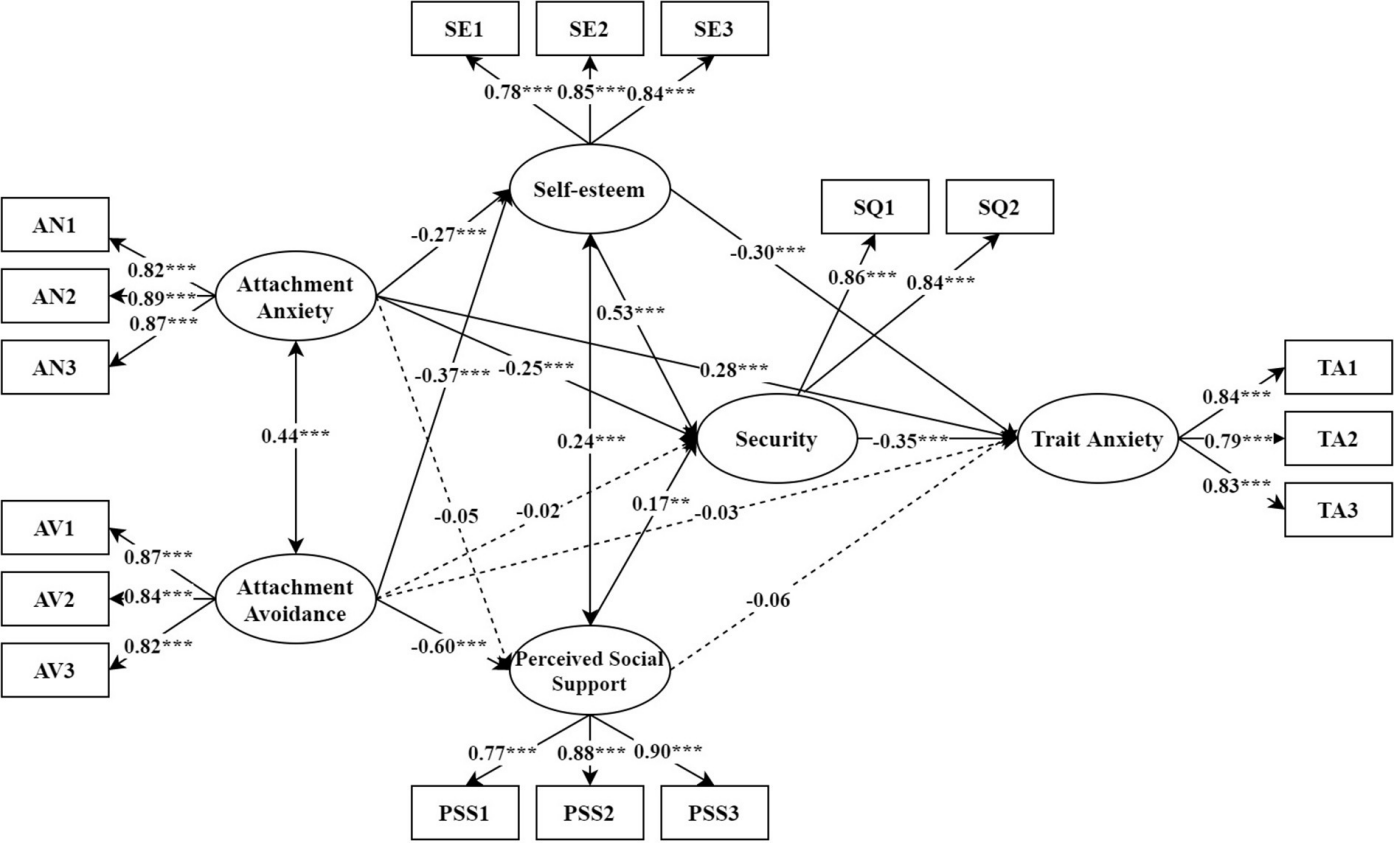
Multiple mediation model. Dashed lines indicate nonsignificant paths. AN1–AN3 = three parcels of attachment anxiety; AV1–AV3 = three parcels of Attachment avoidance; SE1–SE3 = three parcels of self-esteem; PSS1 = family support; PSS2 = friend support; PSS3 = other support; SQ1–SQ2 = two parcels of security; TA1–TA3 = three parcels of trait anxiety. **p < 0.01, ***p < 0.001.

The results of mediation analysis show that the model fits well (χ^2^ = 289.89, *df* = 117, χ^2^/*df* = 2.48, *p* ≤ 0.001; RMSEA = 0.05; CFI = 0.98; TLI = 0.97; SRMR = 0.04). As shown in Figure 2, factor loads of each measurement model range from 0.78 to 0.89, and all are significant at 0.001 level.

As shown in Table 2 and Figure 2, the direct effect of attachment anxiety on trait anxiety is significant (β7), and the total mediating effects in the relationship between attachment anxiety and trait anxiety are significant (β1β12 + β2β13 + β5β11 + β1β9β11 + β2β10β11). Self-esteem (β1β12) and security (β5β11) play mediating roles in the relationship between attachment anxiety and trait anxiety, respectively. Self-esteem and security play serial mediating roles in the relationship between attachment anxiety and trait anxiety (β1β9β11). The direct effect of attachment avoidance on trait anxiety was not significant, but the total mediating effects in the relationship between attachment avoidance and trait anxiety are significant (β3β12 + β4β13 + β6β11 + β3β9β11 + β4β10β11) are significant. Self-esteem (β3β12) plays mediating role in the relationship between attachment avoidance and trait anxiety. Self-esteem and security play serial mediating roles in the relationship between attachment avoidance and trait anxiety (β3β9β11). Perceived social support and security play serial mediating roles in the relationship between attachment avoidance and trait anxiety (β4β10β11).

**Table 2:** Direct and indirect effects of adult attachment on trait anxiety.

**Adult attachment**	**Parameter estimate**	**Bias-corrected CI (95%)**	
	**Effects (SE)**	**Lower**	**Upper**
**Attachment anxiety (Total effects1; H1a)**	0.22 (0.04)^***^	0.17	0.29
AN→ SE→ TA (β1β12; H2a)	0.08 (0.02)^***^	0.05	0.12
AN→ PSS→ TA (β2β13; H3a)	0.003 (0.004)	−0.00	0.01
AN→ Security→ SAS (β5β11; H4a)	0.09 (0.03)^**^	0.04	0.14
AN→ SE4→ Security→ TA (β1β9β11; H4c)	0.05 (0.01)^***^	0.03	0.08
AN→ PSS→ Security→ TA (β2β10β11; H4e)	0.003 (0.003)	0.00	0.01
AN→ TA (β7)	0.28 (0.02)	0.21	0.36
**Attachment avoidance (Total effects2)**	0.26 (0.04)^***^	0.20	0.33
AV→ SE→ TA (β3β12; H2b)	0.11 (0.03)^***^	0.07	0.17
AN→ PSS→ TA (β4β13; H3b)	0.03 (0.03)	−0.02	0.09
AV→ Security→ TA (β6β11; H4b)	0.01 (0.02)	−0.02	0.04
AV→ SE→ Security→ TA (β3β9β11; H4d)	0.07 (0.02)^***^	0.04	0.10
AN→ PSS→ Security→ TA (β4β10β11; H4f)	0.04 (0.01)^**^	0.02	0.06
AV→ TA (β8)	−0.03 (0.05)	−0.11	0.05
**Total effects1 – Total effects2 (H1b)**	0.11 (0.04)	0.05	0.17

*The total effect size is nonstandardized, and the rest is standardized*.

*AN, attachment anxiety; AV, attachment avoidance; SE, self-esteem; PSS, perceived social support; TA, trait anxiety*.

** *p < 0.01*.

*** *p < 0.001*.

By setting up auxiliary variables in the model, we investigated the different internal mechanism of adult attachment predicting trait anxiety. The results show that (non-standard results): attachment anxiety and attachment avoidance have significant differences to predict trait anxiety ((β7 + β1β12 + β2β13 + β5β11 + β1β9β11 + β2β10β11) − (β8 + β3β12 + β4β13 + β6β11 + β3β9β11 + β4β10β11) = 0.11, 95% CI = [0.05, 0.17]); there is no significant difference in the serial mediating effects of self-esteem *via* security ((β1β9β11 – β3β9β11) = −0.01, 95% CI = [−0.03, 0.00]); the serial mediating effects of perceived social support *via* security are significantly different ((β2β10β11) − (β4β10β11) = −0.02, 95% CI =[− 0.03, −0.01]).

## Discussion

This study's descriptive statistical results showed that both attachment anxiety and avoidance positively correlated with trait anxiety. At the same time, effect comparison results showed that the total effect of attachment anxiety predicts trait anxiety is significantly greater than that of attachment avoidance. These results verified H1a and H1b and suggest that there may be different mediating pathways or effects between attachment orientations and relationships. In the following analysis, we further analyzed the mechanism of the difference between attachment anxiety and avoidance and trait anxiety.

Mediating effect analysis found that self-esteem plays mediating role between attachment anxiety and trait anxiety. This result verified H2a and indicated that college students with higher attachment anxiety negatively evaluated themselves. The lower levels of self-esteem were also associated with trait anxiety. In addition, self-esteem also plays mediating role between attachment avoidance and trait anxiety. This result verified H2b and indicated that individuals with higher scores in attachment avoidance might suppress and isolate negative self-cognition by a “defensive mechanism” (33). This result was inconsistent with previous studies with U.S. samples. They found that attachment avoidance was not associated with self-esteem [e.g., (13)]. It was a puzzling and meaningful result that we suspect may be related to differences in the samples collected in the study. It needs to be further deepened and clarified in future studies.

At the same time, we found that perceived social support mediates attachment avoidance and trait anxiety but not between attachment anxiety and trait anxiety. These results verified the H3b, but it was inconsistent with H3a. On the one hand, these results indicated that although college students with high attachment anxiety obtain security by constantly seeking others to establish intimacy, they do not experience the corresponding care and support during this process. These results also explained the intrinsic motivation that leads individuals with high attachment anxiety to adopt a “hyperactivating strategy” (34). Individuals with high attachment anxiety expect to gain support and care through establishing intimate relationships with others, but they do not experience adequate outside support during this process (35). On the other hand, These results indicated that college students with higher scores in attachment avoidance adopt “deactivation strategy” to selectively filter external support and care from others (36). This isolation strategy of intimate relationships leads to higher trait anxiety.

This study found that the mediating effect of security and the serial mediating effect of self-esteem *via* security in the relationship between attachment anxiety and trait anxiety were significant. These results supported H4a and H4c and suggest security plays a critical mediating role in the relationship between attachment anxiety and trait anxiety. In other words, individuals with high attachment anxiety generally lack a sense of security (37), so they easily become a susceptible group to anxiety disorder. At the same time, the serial mediating effect of self-esteem *via* security and the serial mediating effect of perceived social support *via* security in the relationship between attachment avoidance and trait anxiety are significant. These results supported H4D and H4F and indicated that although individuals with high attachment avoidance adopt interpersonal withdrawal strategies (38), they also have a lower sense of security, which explains the core mediating factor of high trait anxiety levels of individuals with attachment avoidance.

## Conclusion

This study reveals the relationship and mediating mechanism between adult attachment and trait anxiety in college students and guided the prevention and intervention of anxiety-related disorders. Specifically, individuals with higher scores in attachment anxiety need to improve their social support skills in interaction with attachment objects while enhancing their self-positive evaluation. While providing adequate social support for individuals with high scores in the attachment avoidance dimension, self-worth training should also be strengthened.

Of course, there are still many deficiencies in this study. First of all, although the mediation model established in this study systematically reveals the relationship between adult attachment and trait anxiety and its internal mediation mechanism, the mediation model fits well. However, the model has many variables and complex relationships, which leads to an insufficient discussion of the relationship between some variables. Secondly, since the structural equation model was built by collecting cross-sectional data in this study, the model results cannot directly support causal inferences. Further analysis can use the longitudinal mediation model to verify and explore the conclusion of this study. In addition, the influence of attachment avoidance on self-representation in the context of Chinese culture needs further research. Thirdly, the relationship between attachment avoidance and self-representation in the context of Chinese culture needs further investigation.

## Data availability statement

The original contributions presented in the study are included in the article/Supplementary material, further inquiries can be directed to the corresponding author/s.

## Ethics statement

The studies involving human participants were reviewed and approved by Academic Ethics Review Committee of Guangdong University of Petrochemical Technology. The patients/participants provided their written informed consent to participate in this study.

## Author contributions

XL designed, wrote, and approved all contributions to the study. PS helped to design the study and edit the manuscrip. LL participated in collecting the data. All authors contributed to the article and approved the submitted version.

## Funding

The research was supported by the Key Scientific Research Platform Project of Guangdong Education Department (2018WTSCX091), the Key Projects of the 13th Five-Year Plan for Education Science of Jiangsu Province (B-a/2020/01/11), the Humanity and Social Science Youth Foundation of Ministry of Education of China (21YJC190014), the Education science Planning project of Guangdong Province (2021GXJK036).

## Conflict of interest

The authors declare that the research was conducted in the absence of any commercial or financial relationships that could be construed as a potential conflict of interest.

## Publisher's note

All claims expressed in this article are solely those of the authors and do not necessarily represent those of their affiliated organizations, or those of the publisher, the editors and the reviewers. Any product that may be evaluated in this article, or claim that may be made by its manufacturer, is not guaranteed or endorsed by the publisher.
